# Key role for lipids in cognitive symptoms of schizophrenia

**DOI:** 10.1038/s41398-020-01084-x

**Published:** 2020-11-12

**Authors:** Dorien A. Maas, Marijn B. Martens, Nikos Priovoulos, Wieteke A. Zuure, Judith R. Homberg, Brahim Nait-Oumesmar, Gerard J. M. Martens

**Affiliations:** 1 Faculty of Science, Centre for Neuroscience, Department of Molecular Animal Physiology, Donders Institute for Brain, Cognition and Behavior, Radboud University Nijmegen, Geert Grooteplein Zuid 26-28, 6525 GA Nijmegen, The Netherlands; 2 Sorbonne Université, Paris Brain Institute – ICM, Inserm U1127, CNRS UMR 7225, Hôpital Pitié-Salpêtrière, Paris, France; 3 Department of Cognitive Neuroscience, Donders Institute for Brain, Cognition and Behavior, Donders Centre for Medical Neuroscience, Radboud University Medical Center, Kapittelweg 29, 6525 EN Nijmegen, The Netherlands; 4 NeuroDrug Research Ltd, Toernooiveld 1, 6525 ED Nijmegen, The Netherlands; 5 Spinoza Centre for Neuroimaging, Meibergdreef 75, Amsterdam-Zuidoost, 1105 BK Amsterdam, The Netherlands

**Keywords:** Molecular neuroscience, Schizophrenia

## Abstract

Schizophrenia (SZ) is a psychiatric disorder with a convoluted etiology that includes cognitive symptoms, which arise from among others a dysfunctional dorsolateral prefrontal cortex (dlPFC). In our search for the molecular underpinnings of the cognitive deficits in SZ, we here performed RNA sequencing of gray matter from the dlPFC of SZ patients and controls. We found that the differentially expressed RNAs were enriched for mRNAs involved in the Liver X Receptor/Retinoid X Receptor (LXR/RXR) lipid metabolism pathway. Components of the LXR/RXR pathway were upregulated in gray matter but not in white matter of SZ dlPFC. Intriguingly, an analysis for shared genetic etiology, using two SZ genome-wide association studies (GWASs) and GWAS data for 514 metabolites, revealed genetic overlap between SZ and acylcarnitines, VLDL lipids, and fatty acid metabolites, which are all linked to the LXR/RXR signaling pathway. Furthermore, analysis of structural T_1_-weighted magnetic resonance imaging in combination with cognitive behavioral data showed that the lipid content of dlPFC gray matter is lower in SZ patients than in controls and correlates with a tendency towards reduced accuracy in the dlPFC-dependent task-switching test. We conclude that aberrations in LXR/RXR-regulated lipid metabolism lead to a decreased lipid content in SZ dlPFC that correlates with reduced cognitive performance.

## Introduction

Schizophrenia (SZ) is a psychiatric disorder with a convoluted etiology and a lifetime prevalence of 0.84%. It is thought that an interplay between genetic, epigenetic, and environmental risk factors is involved in SZ etiology^1^. Symptoms of SZ include positive, negative, and cognitive symptoms^2^. The positive symptoms comprise delusions and hallucinations^3^, the negative symptoms are a loss of typical affective functions^2^, and the most prominent cognitive symptoms of SZ are deficits in attention^4^ and executive functioning^5–7^. There are currently no effective pharmacological treatment strategies that target the negative and cognitive symptoms of SZ^8^. Cognitive symptoms and related changes in the prefrontal cortex (PFC) of SZ patients are already present before disease onset^9^ and contribute negatively to functional outcome^10–13^. Cognitive deficits are found in individuals at high risk to develop SZ^14^ and family members of SZ patients^15^, albeit to a lower degree. The various subregions of the PFC are involved in deficits in specific cognitive domains^16^. For example, although ventro-lateral PFC functioning remains largely unaffected, impaired dorsolateral (dl)PFC-dependent processes are thought to underlie a range of cognitive deficits in SZ^17–19^. In addition, dlPFC activation during the performance of cognitive tasks is decreased in SZ patients^18,20,21^.

Transcriptomic studies on the PFC of SZ patients have increased our understanding of the molecular mechanisms contributing to the PFC-dependent cognitive impairment in SZ. The majority of transcriptomic studies performed on SZ dlPFC (RNA sequencing^22–29^ or microarray^30–32^ analyses) have been conducted on a mix of gray and white matter. However, gray and white matter display discrete gene expression patterns^33^, and therefore investigating the transcriptome of a gray and white-matter mix does not allow the detection of gene expression differences that arise from and are specific to either gray or white matter. One transcriptomic study has been performed on SZ PFC gray matter, but did not specify the PFC subregion that was used^34^. Yet, spatial differences in gene expression patterns exist throughout the cortex^35^ and PFC subregions have distinct contributions to the cognitive deficits in SZ^16^. Only two transcriptomic studies published to date have analyzed solely the gray matter of the SZ dlPFC subregion, with one study reporting differences in the axon guidance pathway^36^ and the other analyzing the expression of only the delta 4-desaturase, sphingolipid 2 (*DEGS2*) gene^37^.

In the current study, we sequenced the transcriptome of the gray matter of dlPFC in SZ and controls. As we found that the differentially expressed genes were enriched in Liver X Receptor/Retinoid X Receptor (LXR/RXR)-mediated lipid metabolism genes, we next investigated whether SZ has a genetic link with lipid metabolism. We indeed identified shared genetic etiology between SZ and among other acylcarnitines, very-low-density lipoprotein (VLDL) lipids, and fatty acid metabolites. Finally, exploratory analyses of structural magnetic resonance imaging (MRI) data were in accordance with a lower lipid content of the dlPFC gray matter in SZ patients as compared to controls and correlated with reduced cognitive performance. Thus, distortions in lipid homeostasis play a key role in the cognitive symptoms of SZ.

## Materials and methods

### Samples and RNA sequencing

Human post-mortem dlPFC brain tissue from four chronic SZ patients and four control individuals was obtained from the Dutch Brain Bank (Amsterdam, The Netherlands). Sample size was based on tissue availability. Sections of 300 µm were obtained in a cryostat (Leica) at −15 °C and two to three punches were collected from different places in the gray matter and in the white matter using a 2.00 mm punch needle (Harris). Punches were frozen on dry ice and stored at −80 °C until RNA isolation using RNeasy lipid tissue mini kit (74804 Qiagen). Isolated RNA was sent for quality control, RNA sequencing, and bioinformatics data analysis to BGI Genomics. Agilent 2100 Bio Analyzer was used to determine RNA quality and RNA integrity numbers of all RNA samples were 6.7 or higher. RNA sequencing was performed using BGISEQ-500 platform generating 6.71 Gb bases per sample. Using hierarchical indexing for spliced alignment of transcripts or HISAT, clean reads were mapped to the reference genome UCSC HG38 with an average of 92.06% mapped reads. Gene expression levels (fragments per kilobase of transcript per million mapped reads) were calculated using RSEM and NOIseq algorithms were then used to determine genes differentially expressed in SZ patients and controls. Significantly differentially expressed genes (probability > 0.8) were used for analysis with the Ingenuity Pathway Analysis software package (Qiagen). RNA sequencing and RNA-sequencing data analysis were performed by researchers that were blinded for disease state. RNA-sequencing data are freely available through https://doi.org/10.6084/m9.figshare.12640460.v1

### Quantitative real-time PCR

For quantitative real-time PCR (qPCR) analysis, per sample 350 μg RNA was treated with DNase I (Fermentas) and cDNA was synthesized using the Revert Aid H-minus first-strand cDNA synthesis kit (Thermo Scientific). cDNA was subsequently diluted 1 : 20 in MilliQ H_2_O and stored at −20 °C until qPCR analysis. qPCR samples contained 2.0 μL diluted cDNA, 0.8 μL 5 μM forward primer, 0.8 μL 5 μM reverse primer, 5 μL SybrGreen mix (Roche), and 1.8 μL MilliQ H_2_O. qPCR was performed with a Rotor Gene 6000 Series (Corbett Life Sciences) using a three-step paradigm with a fixed gain of 8. Fifty cycling steps of 95, 60, and 72 °C were applied and fluorescence was acquired after each cycling step. Primers were designed with NCBI Primer-Blast and synthesized by Sigma (for primer pair sequences, see Supplementary Table S1). Melting temperature was used to check whether a single PCR product was generated and the take off and amplification values of the housekeeping genes (*Ppia* and *Gapdh*) were used to determine the normalization factor with GeNorm^38^ after which normalized mRNA expression levels were calculated. qPCR data were analyzed using Levene’s test for equality of variances and two-tailed independent samples *T*-tests in SPSS Statistics 21. Individual data points and means were plotted using Graphpad Prism 4. Researchers were blinded for disease state during qPCR analysis.

### Shared genetic etiology

Two SZ genome-wide association studies (GWASs) and four metabolite GWAS datasets were used to calculate shared genetic etiology between SZ and metabolite levels. We first calculated shared genetic etiology between 561 metabolites and SZ using previously published SZ GWAS data that was obtained from 33,426 SZ patients from European ancestry^39^. We then replicated the calculation using a second SZ GWAS dataset, namely the GWAS data from 36,989 SZ patients as provided by the Psychiatric Genomics Consortium^40^, which includes the same patients from European descent, but also includes individuals with East-Asian ancestry. The metabolite GWAS data were obtained from Rhee et al.^41^ including 268 metabolite GWASs, Draisma et al.^42^ including 129 metabolite GWASs, Kettunen et al.^43^ including 123 metabolite GWASs, and Ahola-Olli et al.^44^ including 41 cytokine GWASs, and included 2076, 7478, and 24,925 participants of European decent, and 2019 Finnish participants, respectively. Shared genetic etiology was calculated using the freely available program PRSice version 1.23^45^ with PLINK version 1.9 and based on the method of Johnson et al.^46^. Metabolite GWAS data were taken as base samples and SZ GWAS data as the target sample, and correlation results were weighted by the SZ group size. Using PRSice, single-nucleotide polymorphisms (SNPs) were clumped to remove linkage disequilibrium (LD) with an LD threshold of 0.1, a distance threshold of 250 kb, and the 1000 Genomes Project data as genotype reference^47^. A range of SNP significance thresholds was used (*p*_T_ < 0.01, 0.05, 0.1, 0.2, 0.3, 0.4, and 0.5) to calculate shared genetic etiology and the *p*-values obtained using these thresholds were corrected with Bonferroni multiple comparisons correction for the number of metabolites tested.

### Analysis of the dlPFC gray-matter MP-RAGE signal and correlation with task-switching accuracy

The Consortium for Neuropsychiatric Phenomics made available an MRI dataset including 125 healthy individuals (median age = 28 years old, 53% female) and 50 individuals (median age = 37.5 years old, 76% female) diagnosed with SZ or schizoaffective disorder. This dataset includes a T_1_-weighted magnetization prepared–rapid gradient echo (MP-RAGE) sequence (repetition time = 1.9 s, echo time = 2.26 ms, field-of-view = 250 mm, matrix = 256 × 256, slice thickness = 1 mm, 176 slices), as well as cognitive behavioral data from the task-switching test. For details on the dataset, see ref. ^48^. For all MRI analyses, open source code was used. The MP-RAGEs were corrected for B_0_/B_1_ inhomogeneities using the N4 algorithm. A study-specific template of the MP-RAGE scans was created in the common space between the scans with an iterative diffeomorphic warp estimate using the ANTS package^49^. The template was diffeomorphically registered to the MarsAtlas^50^. A segmentation of the dlPFC was extracted from the atlas and projected to each individual scan. The dlPFC regions of interest were corrected at the individual level with a gray-matter mask made with FSL-FAst and the output was visually verified. The average MP-RAGE signal in the dlPFC gray matter of SZ patients and controls was examined. Two linear models were fitted including the average left and right gray-matter dlPFC MP-RAGE signal as the dependent variable and age, sex and group as the independent variables. A retrospective motion-estimate (Average Edge Strength) was also calculated with the homonymous Matlab toolbox^51^ and entered as an independent variable. The analyses were repeated for data acquired at both 3T scanners (Trio, Siemens Healthineers). We then utilized a linear model to analyze the correlation between dlPFC gray-matter MP-RAGE signal and accuracy in the task-switching test in SZ patients accounting for age and motion. For details on the task-switching test, see ref. ^48^. Individual data points and means were plotted using Graphpad Prism 4. Researchers were blinded for disease state during data analysis.

## Results

### RNA sequencing reveals LXR/RXR activation as the top-enriched canonical pathway in gray matter of SZ dlPFC

RNA sequencing was performed on gray matter from dlPFC of four SZ patients and four controls (see Supplementary Table S2 for subject and tissue characteristics). Gene expression density was similar for all samples (Supplementary Fig. S1a) and differential expression analysis showed 132 significantly upregulated genes and 5 significantly down-regulated genes in SZ dlPFC gray matter (Supplementary Fig. S1b). Ingenuity pathway analysis of the significantly differentially expressed genes revealed that “LXR/RXR activation” was the most significantly enriched canonical pathway in the dlPFC of SZ patients (*p* = 3.89E-07 in Benjamini–Hochberg corrected *T*-test; see Table 1 for the top five canonical pathways with statistical values and molecules involved); the other canonical pathways were at least 30 times less enriched. The LXR/RXR pathway regulates cholesterol homeostasis in the brain. The increased abundance of transcripts that are associated with activation of the LXR/RXR pathway indicates a change in cholesterol metabolism in SZ dlPFC gray matter.

**Table 1:** Ingenuity pathway analysis of genes differentially expressed in SZ vs. control dlPFC gray matter.

Canonical pathway	*p*-value (Benjamini–Hochberg corrected)	Genes
LXR/RXR activation	3.89E − 07	*AGT*, *APOC2*, *C4A/C4B*, *IL1RL1*, *S100A8*, *SERPINA1*, *TNFRSF11B*
Complement system	1.15E − 05	*C1QA*, *C1QB*, *C1QC*, *C4A/C4B*
Antigen presentation pathway	1.43E − 05	*HLA-DMA*, *HLA-DQB1*, *HLA-DRB3*, *HLA-DRB5*
PD1-PD-L cancer immunotherapy pathway	5.45E − 05	*HLA-DMA*, *HLA-DQB1*, *HLA-DRB3*, *HLA-DRB5*, *TNFRSF11B*
T-helper cell differentiation	1.71E − 04	*HLA-DMA*, *HLA-DQB1*, *HLA-DRB5*, *TNFRSF11B*

**Fig. 1:**
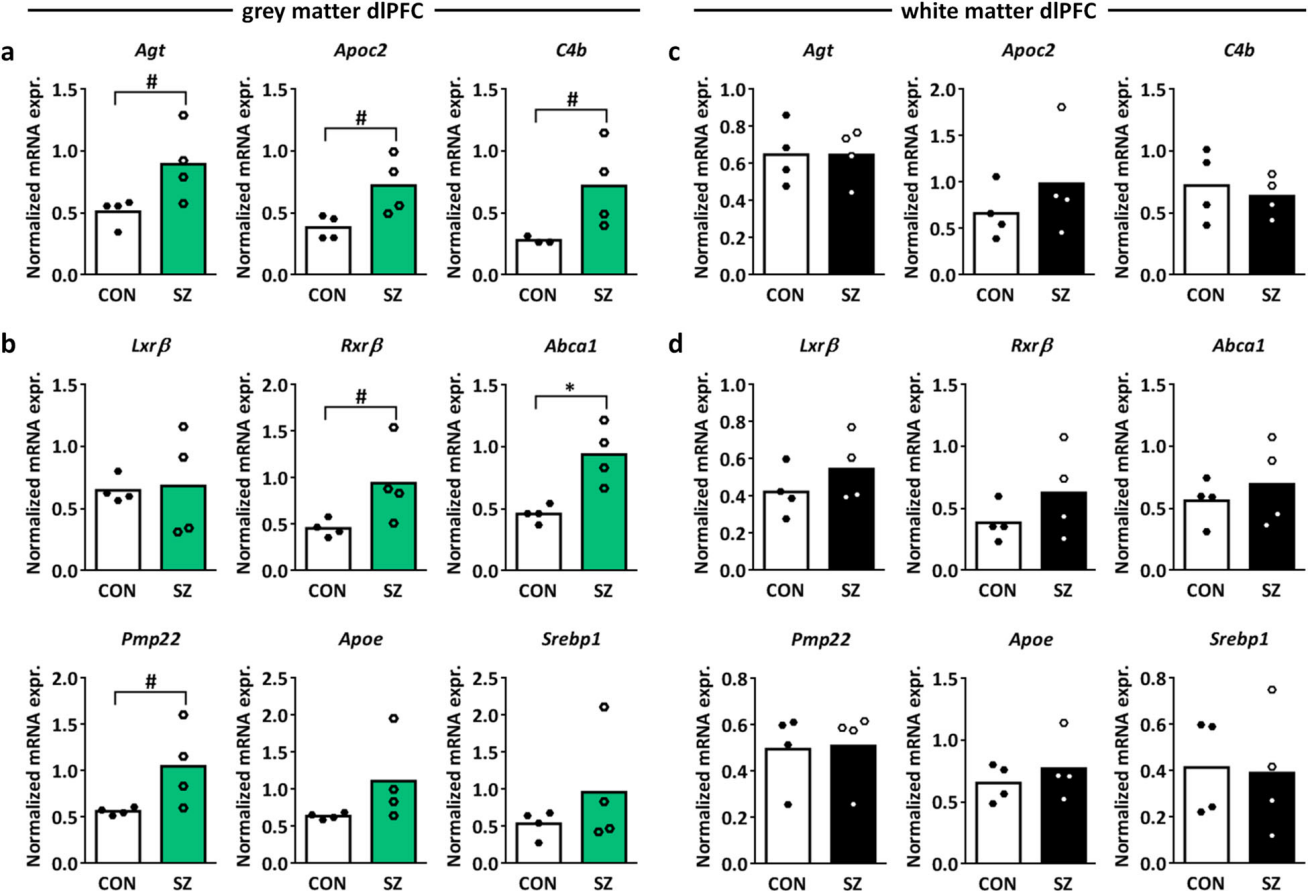
Expression of LXR/RXR-related mRNAs in SZ vs. control dlPFC gray and white matter. **a** Normalized mRNA expression of angiotensinogen (*Agt*), apolipoprotein C2 (*Apoc2*), and complement 4b (*C4b*) in SZ vs. control dlPFC gray matter. These mRNAs are components of the canonical pathway “LXR/RXR activation” in the Ingenuity pathway analysis. **b** Normalized mRNA expression of the LXR/RXR signaling cascade components Liver X receptor β (*Lxr*β), retinoid X receptor β (*Rxr*β), ATP-binding cassette transporter A1 (*Abca1*), peripheral myelin protein 22 (*Pmp22*), apolipoprotein E (*ApoE*), and sterol regulatory element-binding protein 1 (*SREBP1*) in SZ vs. control dlPFC gray matter. **c**, **d** Normalized mRNA expression of the same genes as in **a** and **b** in the white matter of dlPFC. *n* = 4 samples per group, #*p* < 0.1, *p < 0.05 in independent samples *T*-test.

Upregulation of the “LXR/RXR activation” canonical pathway components angiotensinogen (*Agt*), apolipoprotein C2 (*Apoc2*), and complement 4b (*C4b*) in SZ vs. control dlPFC gray matter was confirmed by qPCR (Fig. 1a; independent samples *T*-test *t* = 2.407, *p* = 0.053, df = 6, *t* = 2.673, *p* = 0.056, df=3.986, *t* = 2.155, *p* = 0.083, df=3.059, respectively; see Supplementary Table S1 for primer sequences). We next investigated whether other mRNAs related to LXR/RXR activation were also differentially expressed in SZ dlPFC gray matter. LXRβ is the isoform of LXR that is expressed most abundantly in the brain and forms heterodimers with RXRβ^52^. We found an upregulation of *Rxr*β, but no changes in the mRNA expression of *Lxr*β in the SZ dlPFC gray matter as compared to controls (Fig. 1b; independent samples *T*-test *Rxr*β *t* = 2.202, *p* = 0.070, df = 6, *Lxr*β *t* = 0.156, *p* = 0.885, df = 3.378). The LXRβ/RXRβ pathway activates the transcription factor sterol regulatory element-binding proteins (e.g., SREBP1) and as such stimulates cholesterol and oxysterol efflux from the cell via ATP-binding cassette transporter A1 (*Abca1*), which is regulated by peripheral myelin protein 22 (*Pmp22*)^52–54^. Upon efflux from the cell, cholesterol is packed in the brain in high-density lipoprotein (HDL)-like particles containing apolipoproteins, predominantly apolipoprotein E (ApoE)^52^. *Abca1* and *Pmp22*, but not *Srebp1* and *Apoe*, mRNA expression were upregulated in SZ vs. control dlPFC gray matter (Fig. 1b; independent samples *T*-test *Srebp1* *t* = 1.047, *p* = 0.335, df = 6, *Apoe* *t* = 1.606, *p* = 0.206, df = 3.032, *Abca1* *t* = 3.836, *p* = 0.023, df = 3.538, *Pmp22* *t* = 2.219, *p* = 0.068, df = 6), indicating increased cholesterol efflux in SZ dlPFC gray matter. Notably, in the dlPFC white matter, no changes in LXR/RXR-related mRNA expression were found (Fig. 1c, d), highlighting the importance of studying mRNA expression patterns in the gray and white matter separately.

In addition to the LXR/RXR activation pathway, the canonical pathway analysis revealed significant enrichment of four immune-related pathways in SZ dlPFC gray matter (Table 1), in line with the dysregulation of mRNA expression of genes related to inflammation and the immune system in SZ PFC^25,38^. Furthermore, the top upstream regulator in the Ingenuity pathway analysis was interferon-γ (Supplementary Table S3; *p* = 2.22E − 16) and this and other proinflammatory cytokines are associated with SZ^55–57^.

### Shared genetic etiology between SZ and lipid metabolism

To further investigate the role of lipid metabolism in SZ, we analyzed the shared genetic etiology between SZ and 514 circulating metabolites, including amino acids, nutrients, organic compounds, cytokines, growth factors, and lipids. Following Bonferroni correction (Table 2 and Supplementary Table S4), we found significant overlap between genetic risk for SZ and 35 metabolites (Table 2 and Supplementary Table S4; *p* < 0.05) using the results of the 2018 SZ GWAS study published by the Bipolar and Schizophrenia working group of the Psychiatric Genomics Consortium^39^. Using a second 2014 SZ GWAS dataset provided by the Psychiatric Genomics Consortium^40^, the genetic association between SZ risk and 25 of the 35 metabolites was replicated, and 21 additional metabolites that share genetic etiology with SZ were identified (Table 2 and Supplementary Table S5). Strikingly, the 56 metabolites that share significant genetic etiology with SZ are all related to lipids (except for IP10 and IL16) and fall within three themes: acylcarnitines, VLDL lipids, and fatty acid metabolites. We conclude that disruptions in lipid homeostasis are genetically associated with SZ. The finding that two immune-related metabolites IP10 and IL16 (Table 2) share genetic etiology with SZ is in line with the involvement of the immune system in the disorder.

**Table 2:** Metabolites that share significant genetic etiology with SZ.

Metabolite	Lowest significant *p*-value treshold	Bonferroni- corrected *p*-value	Lowest significant *p*-value treshold	Bonferroni- corrected *p*-value
	SZ GWAS 2018^40^		SZ GWAS 2014^48^	
C5.1.DC^1^	0.1	0.000246	0.05	0.015176
IP10^5^	0.3	0.000997	0.05	0.048767
CH2.DB.ratio^2^	0.1	0.00153	0.1	0.014121
LPE16_0_LIPID^4^	0.001	0.001988	0.001	0.000768
XS.VLDL.TG^3^	0.2	0.002406	0.3	0.039835
C14.1.OH^1^	0.05	0.002534	0.05	0.008502
DB.in.FA^2^	0.1	0.003962	0.1	0.010729
XS.VLDL.P^3^	0.3	0.004685	0.2	0.008044
IDL.C^3^	0.05	0.006454	NA	NA
PC38_2_LIPID^4^	0.3	0.007062	0.2	0.000573
CH2.in.FA^2^	0.2	0.008944	0.1	0.006678
Bis.DB.ratio^2^	0.2	0.009453	0.05	0.018628
DHA^2^	0.4	0.010114	0.05	0.00935
SM.C26.0^4^	0.1	0.010245	0.05	0.002634
XS.VLDL.L^3^	0.05	0.010881	0.2	7.49E-05
TAG54_6_LIPID^4^	0.001	0.016009	0.001	0.009793
SM.OH.C24.1^4^	0.1	0.017865	0.05	0.006078
FAw3^4^	0.05	0.01807	0.05	0.002175
fumarate_maleate_valerat_CMH	0.05	0.019523	0.05	0.001032
Ratio_PC3806_LPC2206_LIPID^4^	0.05	0.023256	NA	NA
IDL.FC^3^	0.4	0.024663	NA	NA
LPC20_3_LIPID^4^	0.3	0.026304	NA	NA
XS.VLDL.PL^3^	0.05	0.027834	0.2	0.00178
XL.VLDL.TG^3^	0.3	0.032077	0.3	0.001178
PC32_0_LIPID^4^	0.1	0.032928	0.1	0.000627
PC.ae.C44.3^4^	0.1	0.034516	NA	NA
IDL.L^3^	0.1	0.034726	NA	NA
S.VLDL.C^3^	0.4	0.035649	0.4	0.047852
IDL.P^3^	0.1	0.036891	NA	NA
lysoPC.a.C20.4^4^	0.3	0.039526	0.2	0.019734
Bis.FA.ratio^2^	0.1	0.040777	0.05	0.005426
S.VLDL.L^3^	0.5	0.0408	NA	NA
GROa	0.001	0.042828	NA	NA
MCP1	0.001	0.045366	0.001	0.001432
LPC22_6_LIPID^4^	0.1	0.048797	NA	NA
Cit	NA	NA	0.05	0.00031
PCB36_4_LIPID^4^	NA	NA	0.05	0.000894
PC38_6_LIPID^4^	NA	NA	0.1	0.002651
CE20_5_LIPID^3^	NA	NA	0.2	0.002948
TAG56_6_LIPID^4^	NA	NA	0.2	0.005974
PC40_6_LIPID^4^	NA	NA	0.3	0.008719
PC.aa.C24.0^4^	NA	NA	0.1	0.010428
TAG56_8_LIPID^4^	NA	NA	0.2	0.012203
IL16^5^	NA	NA	0.05	0.01252
TAG58_10_LIPID^4^	NA	NA	0.3	0.014247
TAG56_6_LIPID^4^	NA	NA	0.3	0.020381
XXL.VLDL.PL^3^	NA	NA	0.3	0.027232
L.VLDL.P^3^	NA	NA	0.05	0.030895
XL.HDL.L^3^	NA	NA	0.5	0.032042
aconitate_CMH	NA	NA	0.1	0.032102
XL.VLDL.L^3^	NA	NA	0.4	0.03703
TAG58_11_LIPID^4^	NA	NA	0.3	0.037887
PC38_4_LIPID^4^	NA	NA	0.4	0.038142
LDL.D^3^	NA	NA	0.001	0.042824
FAw6^4^	NA	NA	0.05	0.044601
ADP_CMH	NA	NA	0.4	0.049512

^1^Acylcarnitines.

^2^Fatty acids.

^3^Cholesterols.

^4^Other lipids.

^5^Immune-related cytokines.

### Lipid content of dlPFC gray matter is lower in SZ than in controls and correlates with reduced accuracy in the task-switching test

**Fig. 2:**
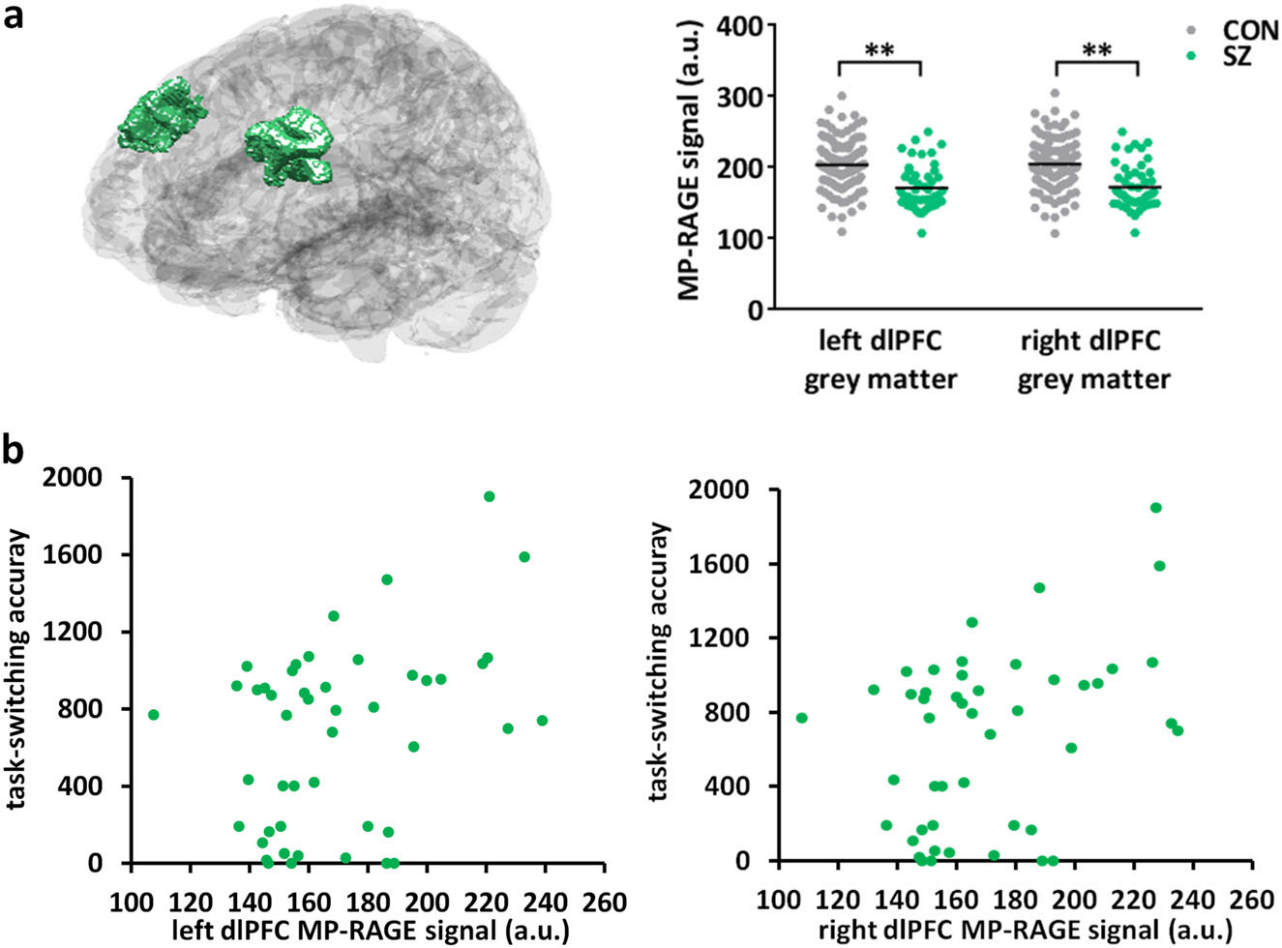
MP-RAGE signal in SZ and control dlPFC gray matter and correlation with task-switching accuracy. **a** Left: schematic representation of the dlPFC gray matter in the left and right brain hemispheres. Right: average MP-RAGE signal from the left and right dlPFC gray matter in SZ *versus* control (CON). **p < 0.001 in a linear model corrected for sex, age, motion, and scanning site. **b** Scatter plot of the accuracy in the task-switching test and the MP-RAGE signal from the dlPFC gray matter in the left and right hemisphere of SZ patients.

We further investigated the effect of the disrupted lipid homeostasis in SZ dlPFC gray matter using a publicly available dataset from the Consortium for Neuropsychiatric Phenomics. This dataset contains amongst others structural MRI scans and performance in the task-switching cognitive test of 50 SZ patients and 125 control individuals^48^. From this dataset, we analyzed the T_1_-weighted MP-RAGE signal. The macromolecular pool in the brain consists mainly of lipids, as illustrated by the typical gray–white matter contrast obtained in T_1_-weighted MRI scans. The T_1_ inversion pulse saturates the free-water pool and the macromolecule pool. Following the saturation, the macromolecular pool quickly relaxes and subsequently accelerates the relaxation of the free-water pool in a process termed magnetization transfer. We hypothesized that a difference in lipid content and thus macromolecular pool would contribute to a change in magnetization transfer. We tested this by comparing the dlPFC gray-matter MP-RAGE signal between SZ patients and controls. We found that the MP-RAGE signal was significantly decreased in the dlPFC gray matter of SZ patients as compared to controls, both in the left and right hemispheres, and accounting for age, sex, motion, and scanning site (Fig. 2a and Supplementary Table S6; linear model left dlPFC estimate = −26.025, *t* = −4.433, *p* < 0.001, right dlPFC estimate = −25.249, *t* = −4.319, *p* < 0.001; Supplementary Fig. S2). These results are in accordance with a lower macromolecular content and thus a lower lipid content of the SZ dlPFC gray matter. Notably, we found a correlation between the accuracy on the dlPFC-dependent task-switching test and the MP-RAGE signal in both the left and right dlPFC accounting for age and motion (Fig. 2b; linear model left dlPFC estimate = 4.286, *t* = 1.946, *p* = 0.0579, right dlPFC estimate = 4.330, *t* = 1.969, *p* = 0.0551). These data are consistent with a lower lipid content of the SZ dlPFC gray matter that correlates with a reduced accuracy in the dlPFC-dependent task-switching test and as such is in line with an important role for a distorted lipid metabolism in the cognitive deficits of SZ.

## Discussion

SZ is a psychiatric disorder with an unknown etiology and its cognitive deficits are associated with the dlPFC. Here we performed RNA sequencing of post-mortem dlPFC gray matter of SZ patients and controls to gain insight into the molecular mechanisms contributing to the cognitive dysfunction in SZ. We found an enrichment of differentially expressed genes in the LXR/RXR activation pathway and validated upregulation of components of the LXR/RXR lipid metabolism pathway in SZ dlPFC gray, but not white, matter. We further revealed shared genetic etiology between SZ and a number of lipid-related metabolites, confirming a genetic link between SZ and lipid metabolism. Finally, the results obtained with the MP-RAGE signals from structural MRI data are in accordance with a decreased lipid content in the dlPFC gray matter of SZ patients and correlated with reduced performance in the task-switching cognitive test.

Gray and white matter have a different cellular composition and function, and distinct transcriptomes^33^. Gray matter of the cortex consists mainly of neurons and glial cells, while white matter consists primarily of myelinated axons. Previous RNA-sequencing studies on mixes of gray and white matter from the dlPFC of SZ patients and controls have shown among others altered abundance of transcripts involved in glucocorticoid signaling^29^, presynaptic function^32^, inflammation^25^, nuclear receptor signaling^23^, synaptic vesicle recycling, transmitter release, and cytoskeletal dynamics^31^. Our RNA-sequencing study on only the dlPFC gray matter confirms the dysregulation of inflammation-related genes in SZ. More importantly, among the differentially expressed dlPFC gray-matter genes the most enriched pathway involved LXR/RXR-mediated cholesterol lipid homeostasis. LXR/RXR-related genes were upregulated in the dlPFC gray matter of SZ patients, but were unaltered in the dlPFC white matter. The previous transcriptomic studies on a mix of SZ dlPFC gray and white matter have likely missed the enrichment of this pathway because of the relatively high contribution of the lipid content of the white matter.

Interestingly, dlPFC gray-matter mRNA expression differences in the axon guidance pathway are known to exist between controls and SZ patients with auditory hallucinations, but not between controls and SZ patients without auditory hallucinations^36^. This highlights that mRNA expression in dlPFC gray matter might differ among subgroups of SZ patients. As in the present study we did not compare subgroups of SZ patients, we may have missed more subtle mRNA expression differences between patients and controls. In addition, for unknown reasons we did not find the previously reported decreased mRNA expression of the SZ-associated *DEGS2* gene in SZ dlPFC gray matter^37^ nor the decreased expression of sodium channel subunit SCN2A, the latter probably due to the fact that we studied a different PFC subregion^34^. Thus, future transcriptomic studies should include SZ patient subgroups and various PFC subregions.

The LXR/RXR pathway is activated by binding of oxysterols to LXR. Oxysterols are metabolites produced during the breakdown of cholesterol and able to cross the blood brain barrier. In the brain, LXRβ forms heterodimers with RXRβ and their activation leads to increased efflux of cholesterol via ABCA1- and PMP22-regulated mechanisms into HDL-like particles containing apolipoprotein, inhibition of cholesterol uptake by the cell and stimulation of fatty acid synthesis^52–54^. We indeed found moderate upregulation of *Rxr*β, *Apoc2*, *Abca1*, and *Pmp22* in SZ dlPFC gray matter as compared to controls. Interestingly, LXR signaling is involved in the development of ventral midbrain dopaminergic neurons^52,58^ and there is a genetic association between PMP22 and SZ^59^. In vitro studies have shown contradictory effects of antipsychotics on LXR signaling in that one study has reported an increased mRNA expression of *Abca1* and *Apoe*^60^, whereas a second study has shown that antipsychotics reduce cholesterol synthesis and export from the endoplasmic reticulum, and do not induce LXR activation^61^. Nevertheless, a disturbance of LXR-mediated cholesterol homeostasis appears to play a role in SZ etiology, but further studies are necessary.

A number of links exist between a distorted lipid homeostasis and SZ. For example, a meta-analysis has revealed that metabolic syndrome in SZ patients, a condition in which cholesterol and triglyceride levels are abnormal, is associated with a high degree of cognitive impairment^62^. Metabolic syndrome also impairs cognition in otherwise healthy individuals^63^. Blood triglyceride levels are correlated with positive symptom severity and blood HDL levels with global functioning of SZ patients^64^. Unmedicated SZ patients have lower total cholesterol, HDL, and apolipoprotein levels^64,65^, and lower short-chain acylcarnitine levels in the blood^66^. Moreover, in SZ patients using antipsychotic medication the occurrence of metabolic syndrome is increased and cholesterol levels are correlated with cognitive impairment^63,67^, implicating a role for peripheral lipid metabolism in brain functioning and cognitive deficits in SZ. In the present study, we find that SZ shares genetic etiology with a number of metabolites, most of which were replicated using a second SZ GWAS study. Among the metabolites that share genetic etiology with SZ, we found an enrichment of acylcarnitines, VLDL lipids and fatty acid metabolites. A previous polygenic risk score analysis has revealed that the severity of cognitive deficits is linked to genetic variations in genes involved in retinoid signaling^68^, a pathway that, similar to the LXR/RXR pathway, is linked to lipid metabolism. Therefore, our results together with this earlier finding highlight a genetic contribution to the observed alterations in lipid homeostasis in SZ that are thus likely not solely caused by antipsychotic treatment.

**Fig. 3:**
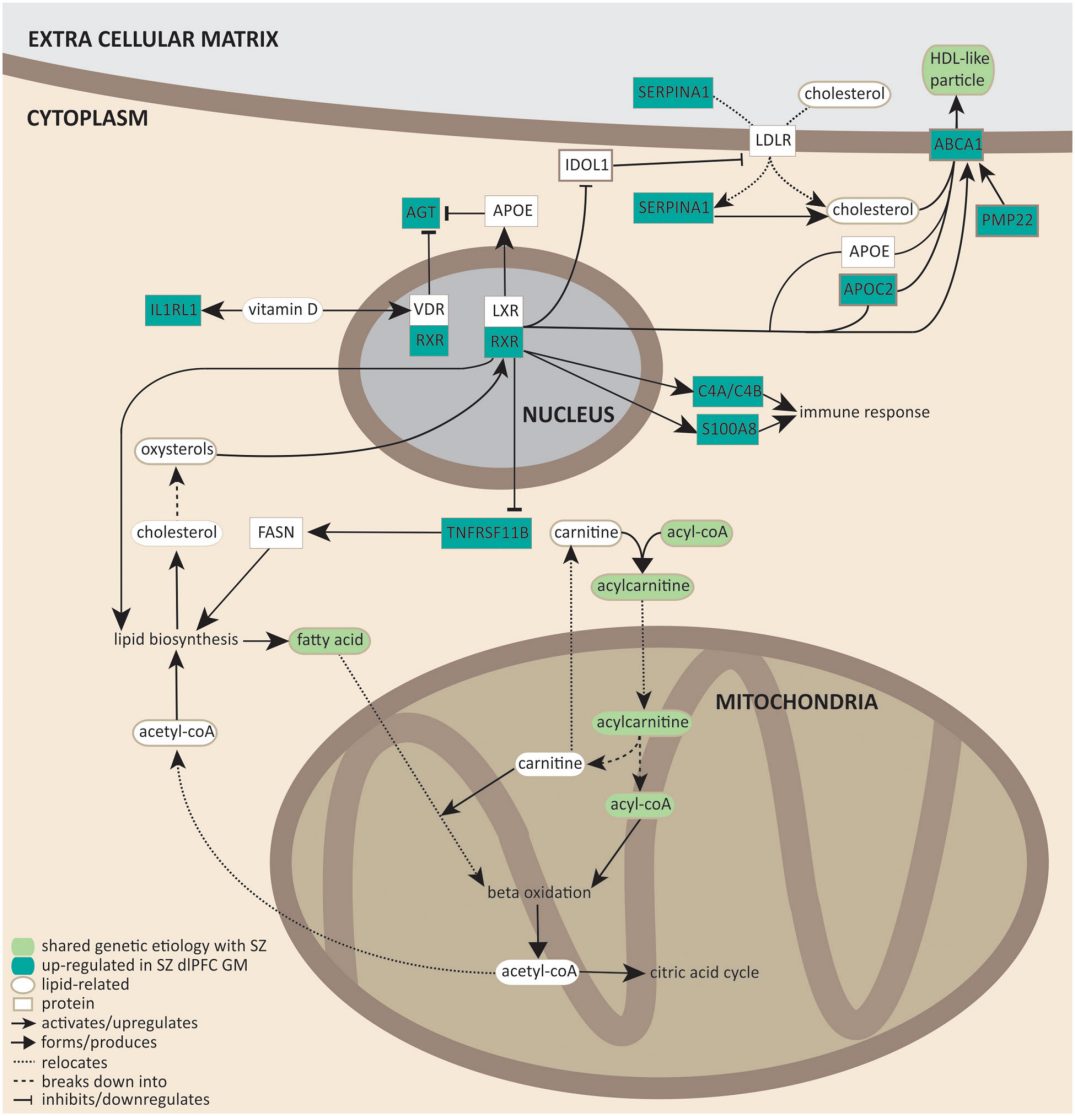
Molecular pathways involving LXR/RXR signaling, acylcarnitines, VLDL lipids, and fatty acid metabolites. Dark green rectangles represent genes upregulated in SZ dlPFC. Light green ovals represent lipid-related metabolites that share significant genetic etiology with SZ. Arrows indicate the nature of molecular interactions (see legend in the figure for details). References^54,68,84–94^ have been used to construct the molecular pathways.

Notably, acylcarnitines, fatty acid production, cholesterol efflux into HDL-like particles, and LXR/RXR activation share a common molecular pathway (Fig. 3). During fatty acid oxidation, unsaturated fatty acids esterify with acyl-CoA to form acylcarnitine that is subsequently transported into the mitochondrial inner membrane. Once inside the inner mitochondrial membrane, acylcarnitines are subjected to β-oxidation, which produces acetyl-CoA that can either enter the citric acid cycle, or is transported to the cytosol where it participates in lipid biosynthesis (fatty acid and cholesterol synthesis). Cholesterol can be transported out of the cell via HDL-like particles. Based on our transcriptomic study and the shared genetic etiology between SZ and several lipid-related metabolites, we conclude that lipid homeostasis involving fatty acid oxidation and cholesterol efflux, production, and transport may well play a role in SZ.

Using a publicly available dataset^48^, we show that the T_1_-weighted MP-RAGE signal is significantly decreased in SZ dlPFC gray matter. The T_1_-weighted MP-RAGE signal creates contrast between gray and white matter, which is thought to be due to magnetization transfer effects, where increased lipid content results in increased signal. Our results are therefore consistent with a decreased lipid content in the gray matter of the dlPFC of SZ patients. This conclusion does not take into account the added effects from differences in spin density or in the relaxation rate of the free-water pool itself to the MP-RAGE signal. However, the relaxation rate of the free-water pool itself is largely homogeneous across the brain^69,70^. Our finding of a decreased lipid content in SZ dlPFC gray matter thus warrants future validation with quantitative magnetization transfer methods. Notably, the decreased MP-RAGE signal in SZ dlPFC gray matter correlates with decreased accuracy in the task-switching test in SZ patients. The task-switching test examines executive functioning and relies on the dlPFC^71–73^. In SZ patients, reduced accuracy^74^ and reaction time differences^75^ in this test have been reported. We find that in SZ altered performance in the task-switching test might arise from a decreased lipid content in the dlPFC.

About half of the dry weight of the brain is attributable to lipids and about 80% of brain lipids are part of myelin sheaths. In SZ PFC, abnormalities in myelination are evident and decreased PFC myelin content contributes to disease symptomatology^76,77^. Furthermore, polyunsaturated fatty acid levels in the blood are correlated with white-matter integrity in frontal regions of the SZ brain^78^, whereas increased LDL levels are associated with white-matter alterations^79^, and white matter, as well as myelin abnormalities in the PFC contribute to cognitive deficits in SZ^76^. Myelin lipids are produced by oligodendrocytes (OLs) and LXRβ-knockout mice show a hypomyelination phenotype, because cholesterol deficiency inhibits OL differentiation and myelination^80,81^. During brain development, LXRβ is also involved in the formation of OL precursor cells^82^ and exerts transcriptional control over myelin-related genes^83^. Therefore, abnormalities in LXR signaling likely contribute to myelin deficits in SZ PFC.

Taken all findings together, we conclude that LXR-driven disturbances in lipid homeostasis are associated with SZ and may mediate the myelination deficits, and as such contribute to the etiology of the cognitive symptoms of SZ.

## Supplementary information

Supplemental material

Supplemental tables S4 and S5

## References

[1] Modai S, Shomron N (2016). Molecular risk factors for schizophrenia. Trends Mol. Med..

[2] Tandon R, Nasrallah HA, Keshavan MS (2009). Schizophrenia, “just the facts” 4. Clinical features and conceptualization. Schizophrenia Res..

[3] Arndt S, Andreasen NC, Flaum M, Miller D, Nopoulos P (1995). A longitudinal study of symptom dimensions in schizophrenia. Prediction and patterns of change. Arch. Gen. Psychiatry.

[4] Fioravanti M, Carlone O, Vitale B, Cinti ME, Clare L (2005). A meta-analysis of cognitive deficits in adults with a diagnosis of schizophrenia. Neuropsychol. Rev..

[5] Lewis R (2004). Should cognitive deficit be a diagnostic criterion for schizophrenia?. J. Psychiatry Neurosci..

[6] Gold JM (2018). Selective attention, working memory, and executive function as potential independent sources of cognitive dysfunction in schizophrenia. Schizophrenia Bull..

[7] Reichenberg A, Harvey PD (2007). Neuropsychological impairments in schizophrenia: Integration of performance-based and brain imaging findings. Psychol. Bull..

[8] Patel KR, Cherian J, Gohil K, Atkinson D (2014). Schizophrenia: overview and treatment options. P T.

[9] Bloemen OJ (2010). White-matter markers for psychosis in a prospective ultra-high-risk cohort. Psychol. Med..

[10] Tamminga CA, Buchanan RW, Gold JM (1998). The role of negative symptoms and cognitive dysfunction in schizophrenia outcome. Int. Clin. Psychopharmacol..

[11] Hintze B, Borkowska A (2015). Associations between cognitive function, schizophrenic symptoms, and functional outcome in early-onset schizophrenia with and without a familial burden of psychosis. Isr. J. Psychiatry Relat. Sci..

[12] Bhagyavathi HD (2015). Cascading and combined effects of cognitive deficits and residual symptoms on functional outcome in schizophrenia - a path-analytical approach. Psychiatry Res..

[13] Joseph J (2017). Predictors of current functioning and functional decline in schizophrenia. Schizophrenia Res..

[14] Mollon J, Reichenberg A (2018). Cognitive development prior to onset of psychosis. Psychol. Med..

[15] Gkintoni E, Pallis EG, Bitsios P, Giakoumaki SG (2017). Neurocognitive performance, psychopathology and social functioning in individuals at high risk for schizophrenia or psychotic bipolar disorder. J. Affect. Disord..

[16] Manoach DS (2003). Prefrontal cortex dysfunction during working memory performance in schizophrenia: reconciling discrepant findings. Schizophrenia Res..

[17] Guo JY, Ragland JD, Carter CS (2019). Memory and cognition in schizophrenia. Mol. Psychiatry.

[18] Ragland JD (2015). Cognitive control of episodic memory in schizophrenia: differential role of dorsolateral and ventrolateral prefrontal cortex. Front. Hum. Neurosci..

[19] Shad MU, Muddasani S, Keshavan MS (2006). Prefrontal subregions and dimensions of insight in first-episode schizophrenia–a pilot study. Psychiatry Res..

[20] Lesh TA (2013). Proactive and reactive cognitive control and dorsolateral prefrontal cortex dysfunction in first episode schizophrenia. NeuroImage. Clin..

[21] Yoon JH (2008). Association of dorsolateral prefrontal cortex dysfunction with disrupted coordinated brain activity in schizophrenia: relationship with impaired cognition, behavioral disorganization, and global function. Am. J. Psychiatry.

[22] Ramaker RC (2017). Post-mortem molecular profiling of three psychiatric disorders. Genome Med..

[23] Corley SM, Tsai SY, Wilkins MR, Shannon Weickert C (2016). Transcriptomic analysis shows decreased cortical expression of NR4A1, NR4A2 and RXRB in schizophrenia and provides evidence for nuclear receptor dysregulation. PLoS ONE.

[24] Tao R (2018). GAD1 alternative transcripts and DNA methylation in human prefrontal cortex and hippocampus in brain development, schizophrenia. Mol. Psychiatry.

[25] Fillman SG (2013). Increased inflammatory markers identified in the dorsolateral prefrontal cortex of individuals with schizophrenia. Mol. Psychiatry.

[26] Birnbaum R (2018). Investigating the neuroimmunogenic architecture of schizophrenia. Mol. Psychiatry.

[27] Davis KN (2016). GAD2 alternative transcripts in the human prefrontal cortex, and in schizophrenia and affective disorders. PLoS ONE.

[28] Hauberg, M. E. et al. Differential activity of transcribed enhancers in the prefrontal cortex of 537 cases with schizophrenia and controls. *Mol. Psychiatry*, 10.1038/s41380-018-0059-8 (2018). 10.1038/s41380-018-0059-8 PMC6222027 29740122

[29] Sinclair D, Fillman SG, Webster MJ, Weickert CS (2013). Dysregulation of glucocorticoid receptor co-factors FKBP5, BAG1 and PTGES3 in prefrontal cortex in psychotic illness. Sci. Rep..

[30] Shao L, Vawter MP (2008). Shared gene expression alterations in schizophrenia and bipolar disorder. Biol. Psychiatry.

[31] Maycox PR (2009). Analysis of gene expression in two large schizophrenia cohorts identifies multiple changes associated with nerve terminal function. Mol. Psychiatry.

[32] Mirnics K, Middleton FA, Marquez A, Lewis DA, Levitt P (2000). Molecular characterization of schizophrenia viewed by microarray analysis of gene expression in prefrontal cortex. Neuron.

[33] Mills JD (2013). Unique transcriptome patterns of the white and grey matter corroborate structural and functional heterogeneity in the human frontal lobe. PLoS ONE.

[34] Dickinson D (2014). Differential effects of common variants in SCN2A on general cognitive ability, brain physiology, and messenger RNA expression in schizophrenia cases and control individuals. JAMA Psychiatry.

[35] Hawrylycz MJ (2012). An anatomically comprehensive atlas of the adult human brain transcriptome. Nature.

[36] Gilabert-Juan J (2015). Semaphorin and plexin gene expression is altered in the prefrontal cortex of schizophrenia patients with and without auditory hallucinations. Psychiatry Res..

[37] Ohi K (2015). DEGS2 polymorphism associated with cognition in schizophrenia is associated with gene expression in brain. Transl. Psychiatry.

[38] Saetre P (2007). Inflammation-related genes up-regulated in schizophrenia brains. BMC Psychiatry.

[39] Genomic Dissection of Bipolar Disorder and Schizophrenia. (2018). Including 28 subphenotypes. Cell.

[40] Consortium SWGOTPG (2014). Biological insights from 108 schizophrenia-associated genetic loci. Nature.

[41] Rhee EP (2013). A genome-wide association study of the human metabolome in a community-based cohort. Cell Metab..

[42] Draisma HHM (2015). Genome-wide association study identifies novel genetic variants contributing to variation in blood metabolite levels. Nat. Commun..

[43] Kettunen J (2016). Genome-wide study for circulating metabolites identifies 62 loci and reveals novel systemic effects of LPA. Nat. Commun..

[44] Ahola-Olli AV (2017). Genome-wide association study identifies 27 loci influencing concentrations of circulating cytokines and growth factors. Am. J. Hum. Genet..

[45] Euesden J, Lewis CM, O’Reilly PF (2015). PRSice: Polygenic Risk Score software. Bioinformatics.

[46] Johnson, T. *gtx: Genetics ToolboX. R package version 0.0.8*. (2013).

[47] Auton A (2015). A global reference for human genetic variation. Nature.

[48] Poldrack RA (2016). A phenome-wide examination of neural and cognitive function. Sci. data.

[49] Avants BB (2011). A reproducible evaluation of ANTs similarity metric performance in brain image registration. NeuroImage.

[50] Auzias G, Coulon O, Brovelli A (2016). MarsAtlas: a cortical parcellation atlas for functional mapping. Hum. brain Mapp..

[51] Zaca D, Hasson U, Minati L, Jovicich J (2018). Method for retrospective estimation of natural head movement during structural MRI. J. Magn. Reson. Imaging..

[52] Courtney R, Landreth GE (2016). LXR regulation of brain cholesterol: from development to disease. Trends Endocrinol. Metab..

[53] Horton JD, Goldstein JL, Brown MS (2002). SREBPs: activators of the complete program of cholesterol and fatty acid synthesis in the liver. J. Clin. Investig..

[54] Zhou Y (2019). PMP22 regulates cholesterol trafficking and ABCA1-mediated cholesterol rfflux. J. Neurosci..

[55] Jemli A (2017). Association of the IFN-gamma (+874A/T) genetic polymorphism with paranoid schizophrenia in Tunisian population. Immunol. Investig..

[56] Paul-Samojedny M (2011). Association study of interferon gamma (IFN-gamma) +874T/A gene polymorphism in patients with paranoid schizophrenia. J. Mol. Neurosci..

[57] Na KS, Jung HY, Kim YK (2014). The role of pro-inflammatory cytokines in the neuroinflammation and neurogenesis of schizophrenia. Prog. Neuropsychopharmacol. Biol. psychiatry.

[58] Theofilopoulos S (2013). Brain endogenous liver X receptor ligands selectively promote midbrain neurogenesis. Nat. Chem. Biol..

[59] Endres, D. et al. Schizophrenia and hereditary polyneuropathy: PMP22 deletion as a common pathophysiological link? *Front. Psychiatry* **10**, 10.3389/fpsyt.2019.00270 (2019). 10.3389/fpsyt.2019.00270 PMC6506456 31118906

[60] Vik-Mo AO, Ferno J, Skrede S, Steen VM (2009). Psychotropic drugs up-regulate the expression of cholesterol transport proteins including ApoE in cultured human CNS- and liver cells. BMC Pharmacol..

[61] Kristiana I, Sharpe LJ, Catts VS, Lutze-Mann LH, Brown AJ (2010). Antipsychotic drugs upregulate lipogenic gene expression by disrupting intracellular trafficking of lipoprotein-derived cholesterol. Pharmacogenomics J..

[62] Bora E, Akdede BB, Alptekin K (2017). The relationship between cognitive impairment in schizophrenia and metabolic syndrome: a systematic review and meta-analysis. Psychol. Med..

[63] MacKenzie NE (2018). Antipsychotics, metabolic adverse effects, and cognitive function in schizophrenia. Front. psychiatry.

[64] Solberg DK, Bentsen H, Refsum H, Andreassen OA (2015). Association between serum lipids and membrane fatty acids and clinical characteristics in patients with schizophrenia. Acta Psychiatr. Scand..

[65] Wu X (2013). The comparison of glycometabolism parameters and lipid profiles between drug-naive, first-episode schizophrenia patients and healthy controls. Schizophrenia Res..

[66] Cao B (2019). Characterizing acyl-carnitine biosignatures for schizophrenia: a longitudinal pre- and post-treatment study. Transl. Psychiatry.

[67] Krakowski M, Czobor P (2011). Cholesterol and cognition in schizophrenia: a double-blind study of patients randomized to clozapine, olanzapine and haloperidol. Schizophrenia Res..

[68] Reay, W. R. et al. Polygenic disruption of retinoid signalling in schizophrenia and a severe cognitive deficit subtype. *Mol. Psychiatry*, 10.1038/s41380-018-0305-0 (2018). 10.1038/s41380-018-0305-0 PMC7156344 30532020

[69] van Gelderen P, Jiang X, Duyn JH (2016). Effects of magnetization transfer on T1 contrast in human brain white matter. NeuroImage.

[70] Gochberg DF, Gore JC (2003). Quantitative imaging of magnetization transfer using an inversion recovery sequence. Magn. Reson. Med..

[71] Premereur E, Janssen P, Vanduffel W (2018). Functional MRI in macaque monkeys during task switching. J. Neurosci..

[72] Dove A, Pollmann S, Schubert T, Wiggins CJ, von Cramon DY (2000). Prefrontal cortex activation in task switching: an event-related fMRI study. Brain Res. Cogn. Brain Res..

[73] Hyafil A, Summerfield C, Koechlin E (2009). Two mechanisms for task switching in the prefrontal cortex. J. Neurosci..

[74] Greenzang C, Manoach DS, Goff DC, Barton JJ (2007). Task-switching in schizophrenia: active switching costs and passive carry-over effects in an antisaccade paradigm. Exp. Brain Res..

[75] Ravizza SM, Moua KC, Long D, Carter CS (2010). The impact of context processing deficits on task-switching performance in schizophrenia. Schizophrenia Res..

[76] Maas DA, Valles A, Martens GJM (2017). Oxidative stress, prefrontal cortex hypomyelination and cognitive symptoms in schizophrenia. Transl. Psychiatry.

[77] Maas DA (2020). Interneuron hypomyelination is associated with cognitive inflexibility in a rat model of schizophrenia. Nat. Commun..

[78] Peters BD (2009). Polyunsaturated fatty acids and brain white matter anisotropy in recent-onset schizophrenia: a preliminary study. Prostagland. Leuk. Essent. Fatty Acids.

[79] Szeszko PR (2014). White matter changes associated with antipsychotic treatment in first-episode psychosis. Neuropsychopharmacology.

[80] Saher G (2005). High cholesterol level is essential for myelin membrane growth. Nat. Neurosci..

[81] Sandoval-Hernandez A, Contreras MJ, Jaramillo J, Arboleda G (2016). Regulation of oligodendrocyte differentiation and myelination by nuclear receptors: role in neurodegenerative disorders. Adv. Exp. Med. Biol..

[82] Xu P (2014). Liver X receptor beta is essential for the differentiation of radial glial cells to oligodendrocytes in the dorsal cortex. Mol. Psychiatry.

[83] Meffre D (2015). Liver X receptors alpha and beta promote myelination and remyelination in the cerebellum. Proc. Natl Acad. Sci. USA.

[84] Remaley AT (2001). Apolipoprotein specificity for lipid efflux by the human ABCAI transporter. Biochem. Biophys. Res. Commun..

[85] Zhu R, Ou Z, Ruan X, Gong J (2012). Role of liver X receptors in cholesterol efflux and inflammatory signaling (review). Mol. Med. Rep..

[86] Longo N, Frigeni M, Pasquali M (2016). Carnitine transport and fatty acid oxidation. Biochim. Biophys. Acta.

[87] Hong C, Tontonoz P (2014). Liver X receptors in lipid metabolism: opportunities for drug discovery. Nat. Rev. Drug Discov..

[88] Gibbons AS (2011). The neurobiology of APOE in schizophrenia and mood disorders. Front. Biosci..

[89] Martorell S (2016). Vitamin D receptor activation reduces angiotensin-II-induced dissecting abdominal aortic aneurysm in apolipoprotein E-knockout mice. Arterioscler. Thromb. Vasc. Biol..

[90] Manna PR, Sennoune SR, Martinez-Zaguilan R, Slominski AT, Pruitt K (2015). Regulation of retinoid mediated cholesterol efflux involves liver X receptor activation in mouse macrophages. Biochem. Biophys. Res. Commun..

[91] Pfeffer PE (2015). Vitamin D enhances production of soluble ST2, inhibiting the action of IL-33. J. Allergy Clin. Immunol..

[92] Subramaniyam D (2010). Cholesterol rich lipid raft microdomains are gateway for acute phase protein, SERPINA1. Int. J. Biochem. Cell Biol..

[93] Goswami S, Sharma-Walia N (2016). Crosstalk between osteoprotegerin (OPG), fatty acid synthase (FASN) and, cycloxygenase-2 (COX-2) in breast cancer: implications in carcinogenesis. Oncotarget.

[94] Robertson KM (2006). Cholesterol-sensing receptors, liver X receptor alpha and beta, have novel and distinct roles in osteoclast differentiation and activation. J. Bone Miner. Res..

