# Supplementary material for: Key role for lipids in cognitive symptoms of schizophrenia
Source: Transl Psychiatry. 2020 Nov 12;10:399. doi: 10.1038/s41398-020-01084-x (PMC7665187; doi:10.1038/s41398-020-01084-x)
Supplement: Supplementary file 1 — Supplemental material [file 41398_2020_1084_MOESM1_ESM.docx]

# Supplementary Material – Maas et al., Key role for lipids in cognitive symptoms of schizophrenia

**Supplementary Table S1 – Primer sequences of primers used for qPCR.**

| Gene | Primer sequence 5' to 3' |
| --- | --- |
| APOC2 forward | ACACTATGGGCACACGACTC |
| APOC2 reverse | GGTCCCCTGGACCTCAAATC |
| C4B forward | CATAGGAGCATGCAGGGGG |
| C4B reverse | TGATGAAGGGCGATGGTCAC |
| AGT forward | GATGGAAGACTGGCTGCTCC |
| AGT reverse | AAGCCCTTCATCTTCCCTTGG |
| LXRb forward | CTGTAAAGGAGGAGGGTCCG |
| LXRb reverse | ATCTGGGATGACCCAGTCTGT |
| RXRB forward | CCTCCTTCTCACACCGATCC |
| RXRB reverse | CCTGGAGAGGGACCGATCAA |
| APOE forward | GGGCCTCTAGAAAGAGCTGG |
| APOE reverse | CTTGGCCTGGCATCCTGC |
| ABCA1 forward | ACTTGGTGGGACGAAACCTC |
| ABCA1 reverse | TACAGGTCTGGGCCTGATGA |
| SREBP1 forward | CTGACCGACATCGAAGGTGA |
| SREBP1 reverse | AAGTGCAATCCATGGCTCCG |

**Supplementary Table S2– Patient and tissue characteristics.**

| Group | Sex | Age | Post-mortem  delay | pH |
| --- | --- | --- | --- | --- |
| Control | Male | 71 | 05:45 | unknown |
| Control | Female | 78 | 07:10 | 6.32 |
| Control | Male | 75 | 07:10 | 6.45 |
| Control | Female | 83 | 06:40 | 6.55 |
| SZ | Male | 67 | 05:45 | 6.29 |
| SZ | Female | 63 | 05:00 | 6.50 |
| SZ | Female | 79 | 04:45 | 6.34 |
| SZ | Female | 55 | 09:50 | 6.82 |

**Supplementary Table S3 - Top upstream regulators in Ingenuity pathway analysis of SZ *versus* control dlPFC.**

| Upstream regulator | P-value |
| --- | --- |
| IFNG | 2.22E-16 |
| Dexamethasone | 1.05E-12 |
| TNF | 1.68E-11 |
| FOS | 2.10E-08 |
| IL1B | 8.21E-08 |

**Supplementary Table S4 - Metabolites that share significant genetic etiology with SZ using SZ GWAS from 2018 ^47^ as target.**Table provided in Supplementary excel file

**Supplementary Table S5 - Metabolites that share significant genetic etiology with SZ using SZ GWAS from 2014 ^48^ as target.**Table provided in Supplementary excel file

**Supplementary Table S6 -** Linear model explaining variance in left and right dlPFC grey matter MP-RAGE signal for both scanning sites.

| Coefficients | Estimate | t-value | p-value | 95% CI |
| --- | --- | --- | --- | --- |
| Left dlPFC grey matter | | | | |
| SZ > Healthy controls | -26.025 | -4.433 | < 0.001 | [-37.616. -14.434] |
| AES | 7226.472 | 2.057 | 0.041 | [291.293, 14161.651] |
| Age | 0.569 | -1.983 | 0.049 | [-1.135, 0.003] |
| Sex | 0.727 | 0.118 | 0.906 | [-12.858, 11.404] |
| Right dlPFC grey matter | | | | |
| SZ > Healthy controls | -25.249 | -4.319 | < 0.001 | [-36.79. -13.707] |
| AES | 6.877.356 | 1.966 | 0.051 | [-28.15, 13782.861] |
| Age | 0.637 | -2.230 | 0.027 | [-1.2, 0.073] |
| Sex | 0.592 | 0.097 | 0.923 | [-12.671, 11.487] |
| Left dlPFC grey matter – scanning site 1 | | | | |
| Schizophrenic > Normal | -34.179 | -2.869 | 0.008 | [-58.542, -9.817] |
| AES | 6342.165 | 0.586 | 0.563 | [-15806.928, 28491.258] |
| Age | 0.104 | 0.137 | 0.892 | [-1.453, 1.662] |
| Sex | -17.290 | -1.130 | 0.268 | [-48.579, 13.999] |
| Right dlPFC grey matter – scanning site 1 | | | | |
| Schizophrenic > Normal | -35.157 | -2.981 | 0.006 | [-59.275. -11.038] |
| AES | 5.164.139 | 0.482 | 0.634 | [-16763.375. 27091.653] |
| Age | 0.001 | 0.002 | 0.998 | [-1.543, 1.54] |
| Sex | -16.044 | -1.059 | 0.298 | [-47.02, 14.932] |
| Left dlPFC grey matter – scanning site 2 | | | | |
| Schizophrenic > Normal | -30.376 | -2.959 | 0.004 | [-50.789. -9.964] |
| AES | 10487.644 | 2.328 | 0.022 | [1530.604. 19444.683] |
| Age | 0.699 | -1.693 | 0.094 | [-1.521, 0.122] |
| Sex | 0.164 | 0.019 | 0.985 | [-17.327, 16.998] |
| Right dlPFC grey matter – scanning site 2 | | | | |
| Schizophrenic > Normal | -29.820 | -2.896 | 0.005 | [-50.297, -9.344] |
| AES | 10553.526 | 2.336 | 0.022 | [1568.473, 19538.579] |
| Age | 0.684 | -1.651 | 0.103 | [-1.508. 0.14] |
| Sex | 0.843 | 0.097 | 0.923 | [-18.059, 16.373] |

**
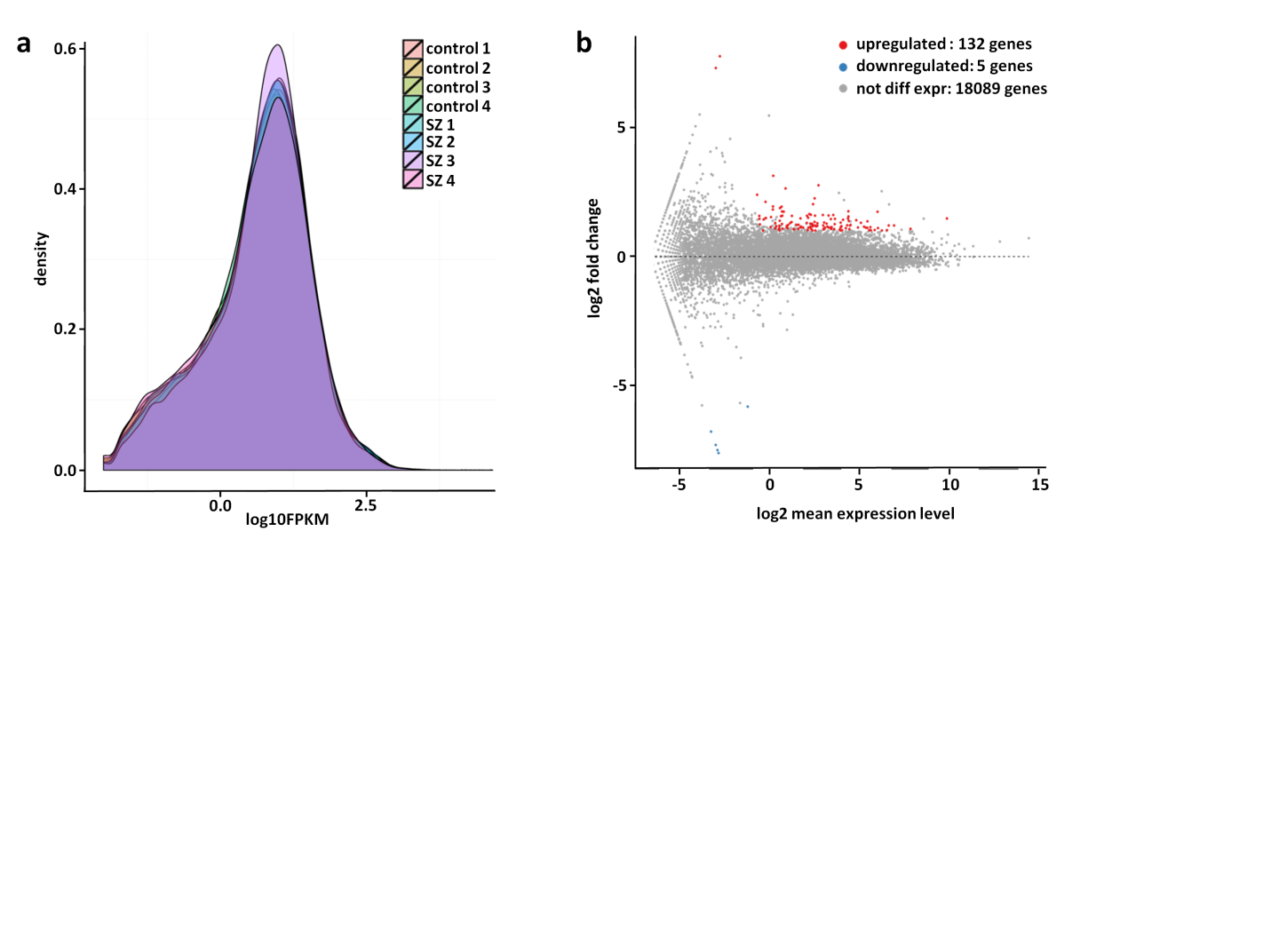
**

**Supplementary Figure S1 – mRNA expression profiles of SZ *versus* control dlPFC. (a)** Gene expression density profiles over FPKM for all samples. **(b)** Scatter plot for the log2 fold change versus the log2 mean FPKM expression level for all transcripts. Red and blue dots represent significantly (|fold change| > 1 and probability > 0.8) up- and downregulated transcripts, respectively. Grey dots represent transcripts that were not significantly differentially expressed.

**
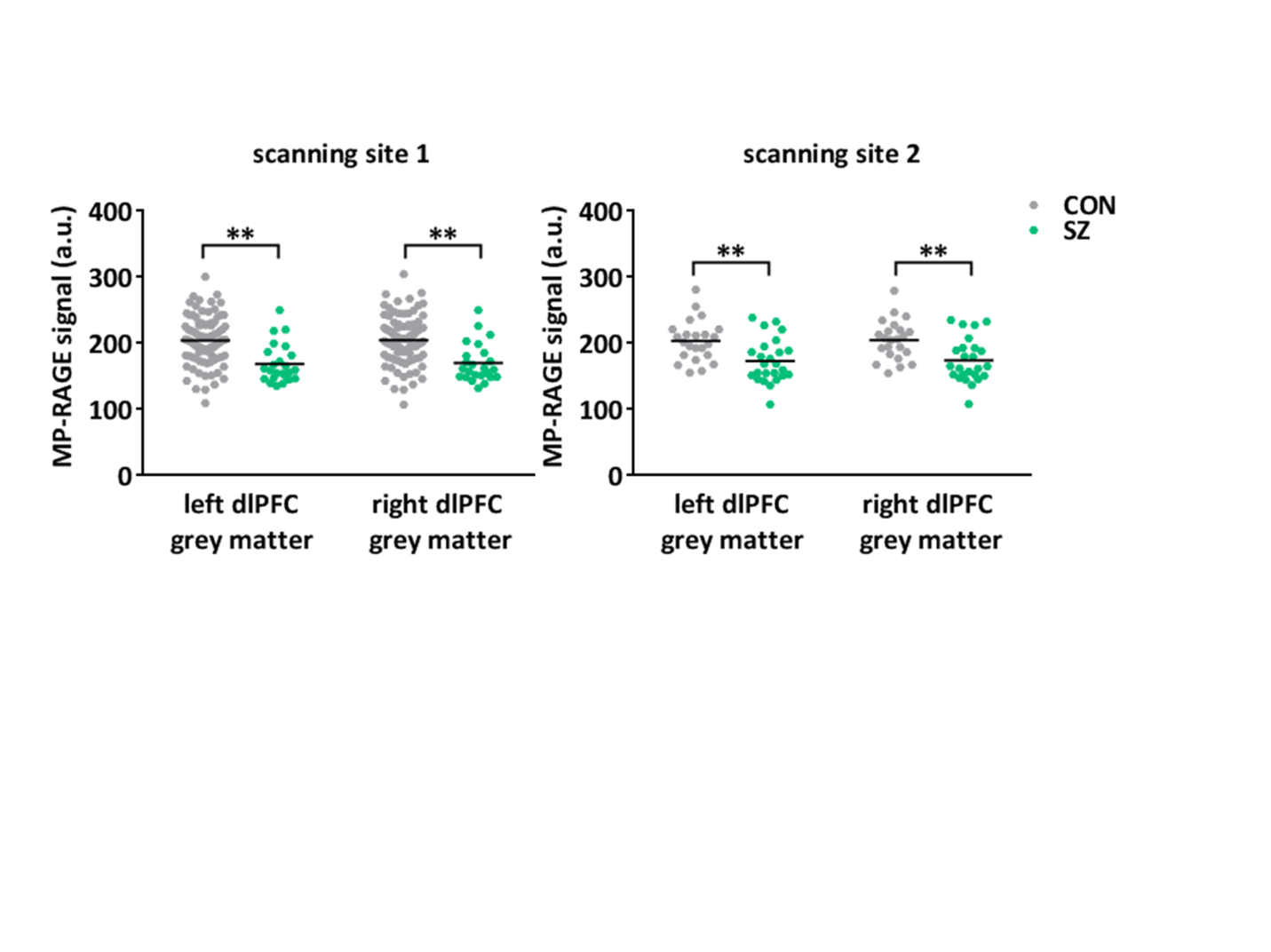
**

**Supplementary Figure S2 – Average MP-RAGE signal in SZ *versus* control dlPFC grey matter in the left and right hemispheres and at both scanning sites**. **p<0.001 in a linear model.
